# Does Storage under Gene Bank Conditions Affect Seed Germination and Seedling Growth? The Case of *Senecio morisii* (Asteraceae), a Vascular Plant Exclusive to Sardinian Water Meadows

**DOI:** 10.3390/plants9050581

**Published:** 2020-05-02

**Authors:** Alba Cuena-Lombraña, Martina Sanna, Marco Porceddu, Gianluigi Bacchetta

**Affiliations:** 1 Department of Life and Environmental Sciences, Centre for the Conservation of Biodiversity (CCB), University of Cagliari, Viale Sant’Ignazio da Laconi, 11-13, 09123 Cagliari, Italy; porceddu.marco@unica.it (M.P.); bacchet@unica.it (G.B.); 2 Sardinian Germplasm Bank (BG-SAR), Hortus Botanicus Karalitanus (HBK), University of Cagliari, Viale Sant’Ignazio da Laconi, 9-11, 09123 Cagliari, Italy; m.sanna17@studenti.uniss.it

**Keywords:** ex situ conservation, long-term storage, seed banking, seed viability, survival, restoration programs

## Abstract

Understanding seed viability under long-term storage conditions provides basic and useful information to investigate the effectiveness of seed banking. Besides the germination success, seedling establishment is also an important requirement, and a decisive step to ensure plant propagation. We used comparative data of germination, seedling growth, and survival percentage between fresh and 10-years-stored seeds of *Senecio morisii*, a narrow endemic and vulnerable species of Sardinia (Italy), in order to evaluate if differences exist in these traits between fresh and 10-years-stored seeds. Stored seeds showed higher germination percentages than fresh ones, whereas seedling growth and survival did not present significant differences between them, except for seedling growth in plants produced from seeds germinated at 25 °C. This study allowed us to assess if seeds of *S. morisii* were able to germinate under controlled conditions, and if they maintained their viability and germination capacity for at least 10 years of long-term storage in the seed bank. In addition, the high seedling survival detected in both fresh and stored seeds suggests that stored seeds of *S. morisii* can be used to support reinforcement or reintroduction actions when fresh materials are not available.

## 1. Introduction

Seed banking, as an integral part of the ex situ conservation, has a pivotal role in safeguarding plant species for long times in order to avoid loss of genotypes from populations. Ex situ conservation is complementary to in situ methods, serves as a source of material for in situ conservation actions [1,2,3] and, for some species, it might be the only option for their preservation [4,5]. About 45% of Europe’s vascular plants are considered threatened, according to IUCN criteria [6]. Furthermore, Target 8 of the Global Strategy for Plant Conservation (GSPC) calls for “at least 75% of threatened plant species in ex situ collections, preferably in the country of origin, and at least 20% available for recovery and restoration programmes” [7,8]. Therefore, extra attention is necessary to ensure the ex situ conservation of endangered plants in seed banks and to develop effective germination and multiplication protocols for these species.

Seed banking is a particularly important conservation strategy for species with orthodox seeds (desiccation-tolerant, *sensu* [9]). These seeds can be gradually dried at 15 °C and 15% of relative humidity (RH), in order to reach ca. 3%–5% of internal seed moisture content, and then stored safely at temperature near to −18 and −25 °C [10]. Seed storage conditions maintain germplasm viability for several years, but even under suitable conditions, viability might decline over time [11]. Seeds of some species can remain viable for hundreds of years, while seeds from other species can only survive for few years [12,13]. For this reason, it is necessary to assess periodically the viability of seeds stored in seed banks. International guidelines and standards indicate that the viability of stored collections should be tested every five or 10 years [12,14,15,16,17]. Loss of seed viability and seed dormancy due to seed ageing have been found in many species [13,18,19,20], and are caused by metabolic changes and DNA deterioration [21]. 

Seed germination studies have also been suggested to be performed in order to evaluate the effective viability of stored seeds [22]. For instance, seedling establishment and survival are vital to guarantee the persistence and multiplication of plant populations, and the successive effectiveness of restoration strategies [23]. Consequently, knowledge of the germination process, seedling establishment, and survival of threatened species are important for conservation practitioners, enabling them to produce plants and to increase the chance for establishment of self-sustaining populations [24,25]. 

In this paper, we studied the germination capacity of a threatened and narrow endemic species of Sardinia (Italy), *Senecio morisii* J.Calvo & Bacch. Existing within the genus *Senecio* L. (Asteraceae), one of the largest genera of flowering plants, almost cosmopolitan and occurring in all the five regions with a Mediterranean climate [26], this species forms part of the *Senecio doria* L. group, a species complex of perennial herbs from Europe, western and central Asia, and northwestern Africa [27]. *S. morisii* was recently described from central-eastern Sardinia; it is a hygrophilous species which grows on water meadows or watersides with calcareous soils (limestones, travertines, and conglomerates) [27]. In particular, it lives along the edges of streams in woods of *Ostrya carpinifolia* Scop. accompanied by *Taxus baccata* L. and *Ilex aquifolium* L., between elevations of 700–1200 m a.s.l. [26]. *S. morisii* flowers from late May to July; the ligulate florets produce achenes, subcylindrical and glabrous, at the end of July, and no information about its germination traits is currently available. Only six populations are currently known [27,28] and it was assessed as Vulnerable (VU) in the IUCN Italian Red List [29].

Comparative data of seed germination, seedling growth, and survival percentage between fresh and 10-years-stored seeds of *S. morisii* were used to address the following questions: (1) have fresh and stored seeds the ability to germinate?; (2) if yes, are there differences in germination between fresh and stored seeds?; and (3) are there differences in seedling growth and survival between fresh and stored seeds?

## 2. Results

### 2.1. Seed Germination

**Figure 1:**
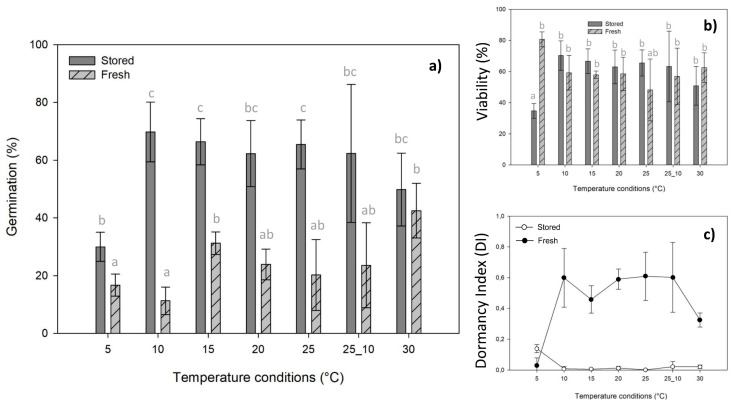
(**a**) Final germination percentages (FGPs) achieved at the end of the germination tests for each seed conditions (stored and fresh). (**b**) Percentage of seed viability at the end of the germination tests. Seed viability was calculated as sum of germinated seeds and filled seeds. Post hoc pairwise *t*-test comparisons were carried out for each germination temperature, and bars with different letters (a, b, c) indicate significant (*P* < 0.05) differences. (**c**) Dormancy index (DI) by temperatures of stored and fresh seeds at the end of germination tests.

Generalized linear models (GLMs) show that seed germination under different conditions (stored or fresh) and incubation temperatures were significantly different (*P* < 0.001, Table S1), also according to the two-way interaction (*P* < 0.01, Table S1). Seeds stored (SS) for 10 years showed high germination percentage at all tested temperatures (Figure 1a). In general, all the SS achieved more than 50% of final germination percentages (FGPs), except at 5 °C (30.00 ± 5.00%) and at 30 °C (49.85 ± 12.63%) (Figure 1a). FGPs ranging from 60% to 70% at the temperature range from 10 °C to 25 °C including alternate temperature, were as follows: the highest FGPs were detected at 10 °C (69.79 ± 10.33%), followed by 15 °C (66.39 ± 8.02%), 25 °C (65.44 ± 8.48%), 25/10 °C (62.36 ± 23.90%), and 20 °C (62.26 ± 11.42%). On the other hand, fresh seeds (FS) did not reach 50% of germination at any incubation temperature (Figure 1a). The highest FGP was achieved at 30 °C with 42.50 ± 9.47%, followed by germination at 15 °C (31.23 ± 3.90%), 20 °C (23.90 ± 5.34%), 25/10 °C (23.59 ± 14.71%), and 25 °C (20.22 ± 12.30%). The FS showed a FGP lower than 20% at 5 and 10 °C. 

Regarding the viability (Figure 1b), no significant differences (*P* > 0.05, Table S2) were detected by GLM between seed conditions (SS and FS) and the two-way interactions (seeds and temperature conditions). While incubation temperatures were slightly significantly different (*P* = 0.05, Table S2), the subsequent post hoc highlighted significant differences at 5 °C (*P* < 0.05, Figure 1b). Analyzing the dormancy index (DI), values were particularly high in FS (Figure 1c). DI in FS at temperature range from 10 °C to 30 °C was higher than the threshold value of dormancy > 0.4, while only the DI value for 5 °C was less than 0.2. For SS, DI was less than 0.2 in all tested temperature conditions (Figure 1c).

### 2.2. Seedling Growth and Survival Percentage

**Figure 2:**
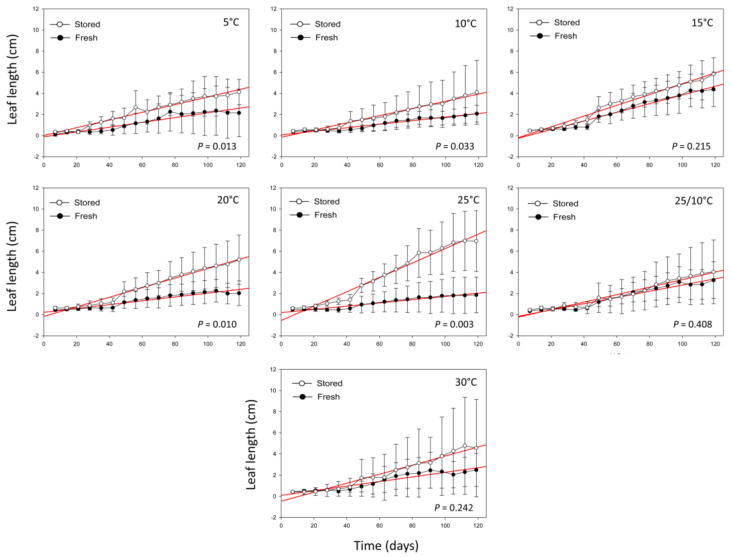
Seedling growth, expressed as leaf length measured, by plants obtained from germinated seeds in each incubation temperature and seed condition (stored and fresh). Measures of leaf length are expressed by time unit, according to the following equation: unit = cm × day^−1^. Red lines correspond to linear regressions.

The leaf length of seedlings belonging to SS and FS (Figure 2), measured during 120 days under greenhouse conditions at 18 °C, did not show very significant differences (*P* > 0.01, Table S3), except for the seedlings obtained from germinated seeds at 25 °C (*P* = 0.003, Table S3). The highest leaf lengths for SS were found in seedlings derived from germinated seeds at 25 °C (6.58 ± 2.96 cm), followed by 15 °C (5.95 ± 1.62 cm), and 20 °C (5.38 ± 2.37 cm). Regarding the FS, the highest leaf lengths were detected in seedlings from germinated seeds at 15 °C (4.11 ± 1.90 cm) and 25/10 °C (2.85 ± 1.37 cm).

**Figure 3:**
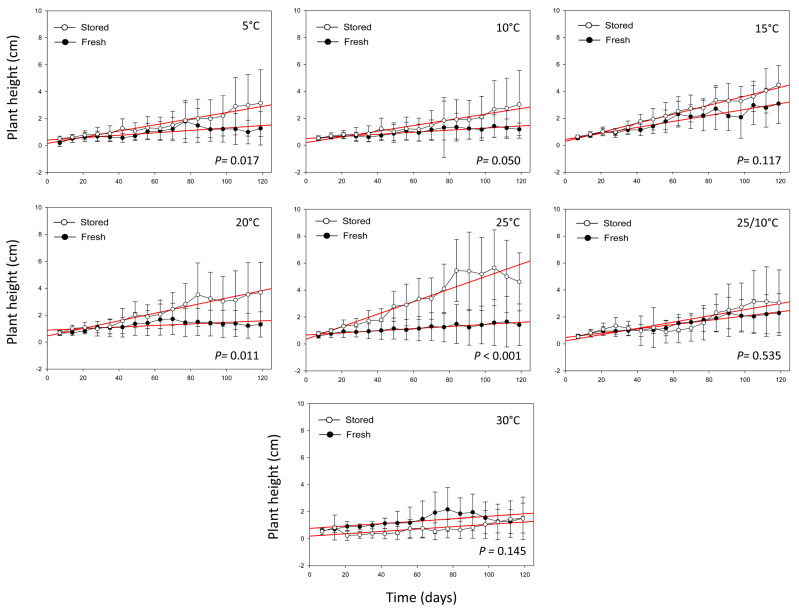
Seedling growth, expressed as plant height measured, by plants obtained from germinated seeds in each incubation temperature and seed condition (stored and fresh). Measures of plant height were expressed by time unit, according to the following equation: unit = cm × day^−1^. Red lines correspond to linear regressions.

Plant height (Figure 3) did not present very significant differences (*P* > 0.01, Table S3) between seedlings obtained from SS and FS, except for the temperature of 25 °C (*P* = 0.001, Table S3). The highest plant heights in SS were found in seedlings obtained from germinated seeds at 25 °C (4.73 ± 2.19 cm) and 15 °C (4.19 ± 0.99 cm), followed by 20 °C (3.54 ± 2.40 cm) and alternating temperatures 25/10 °C (3.46 ± 2.75 cm). Regarding FS, the highest plant heights were found for seeds germinated at 15 °C (2.33 ± 1.30 cm) and alternating temperatures 25/10 °C (1.94 ± 0.91 cm).

The survival percentage between seedlings obtained from SS and FS did not show significant differences (*t* = –0.0432; *P* = 0.966, Table S4). The highest survival percentages (100%) were found for the SS from germinated seeds at 15, 20, and 25 °C while, for the FS, they were found in seedlings from germinated seeds at 10 and 15 °C (Table S4).

## 3. Discussion

*Senecio morisii* is a vulnerable plant species endemic to Sardinia, for which *ex situ* conservation has become a necessary strategy. Knowing and understanding the complex elements that control seed longevity and germination are therefore of major ecological and conservational importance [30]. This is the first attempt, to our knowledge, to investigate the seed germination of fresh and stored seeds of *S. morisii*. The results show that both fresh and stored seeds are able to germinate under controlled conditions. In addition, the high viability detected in seeds stored for 10 years under seed banking conditions at −25 °C allows us to suggest their use in the absence of fresh material for management actions, like translocations and/or population reinforcements. Especially in the case of endemic and/or threatened plants, which may present problems related to the poor availability of material for plant propagation, seed banks can be decisive sources of material for translocations [31].

Seeds of *S. morisii*, collected in 2007 and then stored at −25 °C for 10 years, showed a broader germination temperature and higher germination rates, when compared with the fresh ones collected in 2017. Stored seeds presented a germination of approximately 70%, while the germination of fresh seeds of *S. morisii* was less than 42%. Compared with other species of the genus *Senecio*, this species presented similar requirements for the germination. For example, *S. malacitanus* Huter, that is native to the southwestern Mediterranean Basin, also germinated with high percentages in a wide range of temperatures, from 5 to 30 °C [32]. Furthermore, studies of *S. vulgaris* L. show that germination was also favored at 20 °C and higher temperatures [33,34]. The highest germination percentage of fresh seeds of *S. morisii* was found at 30 °C. This could be an adaptation derived from the dry conditions that occur in natural habitats of Mediterranean hygrophilous species. Usually, these species germinate during the driest season in order to avoid that seedling development would coincide with the occurrence of flooding, usually during cool seasons (i.e., autumn and winter). Specifically, after dispersal in early summer, *S. morisii* seeds experience a dry period (from July to September), characterized by high temperatures and low flow of the rivers or springs where this species grows.

To better understand the reason for the lower germination detected (less than 50%) in fresh seeds, we used seed viability percentage and DI as indexes. Firstly, the viability did not present statistical differences between stored and fresh seeds (except at 5 °C). Therefore, seed viability related to idiosyncratic causes influenced by the particular conditions of each year had not caused differences on germination, despite several studies that proved the interannual germination plasticity related to interannual climatic fluctuations [35,36]. Moreover, according to [2] and [13], high DI values (>0.4) of fresh seeds indicated the presence of some kind of dormancy. Thus, our results allow us to hypothesize that *S. morisii* fresh seeds may be partially dormant. Further studies are needed to evaluate this and define the type of dormancy [37]. Unfortunately, the extreme ability of the achenes to disperse by wind, the limited population size, and other threats of this species, such as the low number of flowering individuals due to overgrazing, were a limit for ensuring the availability of material for these experiments. 

Even if seed germination was high at a wide range of temperatures, we detected that seedlings showed better growth performance and survival percentages when coming from germinated seeds at the incubation temperatures of 20 and 25 °C. This is in accordance with [38], who demonstrated that plant developmental processes are complex, but strongly dependent on incubation conditions. 

Seed storage under gene bank conditions for 10 years had not affected seedling growth and survival percentage in the first stage of plant development. Indeed, seed viability and seedling establishment (survived after an experimental period of 120 days) were similar across the stored and fresh seeds. Interestingly, percentages of seedling survival were higher than 50% in all conditions. 

These results provide information on seed germination and seedling establishment of this narrow endemic species, offering basic but useful information for the conservation efforts of *S. morisii*, where translocation actions are recommended due to its restricted distribution range (limited to six populations) and the intensive grazing that affects the conservation status of this vulnerable species. 

## 4. Materials and Methods

### 4.1. Seed Lots Details

During July 2017, mature seeds (achenes) of *S. morisii* were sampled in the population of Montarbu, located in Seui (C-E Sardinia) at mean altitude of 924 m a.s.l. Fresh seeds (FS) were collected from at least 30 individuals. Afterwards, they were cleaned manually, removing any visually damaged seeds, and stored for two weeks until the start of the germination experiments at room temperature (ca. 20 °C and 40% of R.H.). Stored seeds (SS) were collected in July 2007, also from the population of Montarbu, and were stored at −25 °C in the Sardinian Germplasm Bank (BG-SAR) following the international standards for long-term storage [14,15].

### 4.2. Seed Germination Trials

Once the stored seeds were removed from −25 °C, their humidity was progressively recovered at room temperature (ca. 20 °C and 40% RH). Subsequently, germination tests of both stored and fresh seeds were carried out together and started within two weeks after fresh seed collection. Three replicates of 20 seeds for each experimental condition were used. The seeds were sown on the surface of 1% agar water in plastic Petri dishes and incubated in controlled conditions under a range of constant temperatures (5, 10, 15, 20, 25 and 30 °C), and under an alternating temperature regime (25/10 °C), with a photoperiod of 12/12 h light/darkness. In the alternating temperature regime, the higher temperature coincided with the 12-h light period. Seeds were incubated in growth chambers (Sanyo MLR-351; SANYO Electric, Osaka, Japan), each one equipped with white fluorescent lamps (FL40SS.W/37 70–10 μmol m^−2^ s^−1^). Germination was recorded three times a week, and seeds were considered germinated when an emerging radicle was longer than 1 mm. Afterwards, germinated seeds were transferred to other Petri dishes to allow seedling development. For each germination experiment, the final germination percentage (FGP) was calculated, which was determined as the mean of the three replicates (± SD), considering the total number of filled seeds (empty seeds were omitted). At the end of the germination tests (for a minimum of 90 days), when no additional germination occurred for two consecutive weeks, seed viability was estimated. This was calculated as the number of germinated seeds plus the number of filled seeds from the cut test [10] and expressed as a percentage of the total [13,39]. In addition, the evaluation of dormancy status was calculated using the equation from [2]: Dormancy index (DI) = 1 − [germinated seeds (%)/seed viability (%)]. An index > 0.4 was used as the threshold value to indicate dormancy [2,13]. 

### 4.3. Seedling Growth and Survival Percentage

Once seeds germinated, seedlings with cotyledons were moved in a substrate of 1% agar water into other Petri dishes for two weeks. Therefore, we randomly selected ten germinated seeds by temperature and by experimental conditions (SS and FS) to be transplanted and separately measured according to each temperature tested. Developed seedlings were transplanted in potting soil in a greenhouse using peat moss as substrate, and incubated simulating a day/night cycle (12/12 h light/darkness) under a constant temperature (ca. 18 °C), and watered three times a week using the same quantity of water. Seedling growth after transplantation was evaluated and the growth rate was estimated as an increment of morphometric variables of individual plants. Concretely, we used leaf length and plant height, by time unit, in accordance with the following equation (unit = cm × day^−1^). Measurements were made once per week. In addition, the survival was monitored and recorded during the total experimental period (120 days).

### 4.4. Statistical Analyses

Generalized linear models (GLMs) were used to evaluate differences in the germination responses (FGP) and seed viability among seed conditions (SS or FS) and temperatures of incubation tested. Significant differences identified by GLMs were then analyzed by a post hoc pairwise comparison *t*-test (with Bonferroni adjustment). For analyzing the FGP and seed viability, we used a log link function and quasibinomial error structure. In addition, we evaluated the differences between means of seedling growth measures (leaf length and plant height by time unit) and survival percentages by seed conditions (SS or FS), using *t*-test (for normally distributed variables) and Mann–Whitney test (for non-normally distributed variables). All statistical analyses were performed using R v. 3.2.2 [40].

## Supplementary Materials

The following are available online at https://www.mdpi.com/2223-7747/9/5/581/s1, Table S1: GLM results of seed final germination percentage (FGP); Table S2: GLM results of viability; Table S3: Mann–Whitney test results for leaf length and plant height measures; Table S4: Survival data of seedlings at the finish of the experiments (120 days).
