# Supplementary material for: Does Storage under Gene Bank Conditions Affect Seed Germination and Seedling Growth? The Case of Senecio morisii (Asteraceae), a Vascular Plant Exclusive to Sardinian Water Meadows
Source: Plants (Basel). 2020 May 2;9(5):581. doi: 10.3390/plants9050581 (PMC7284729; doi:10.3390/plants9050581)
Supplement: Supplementary file 1 [file plants-09-00581-s001.pdf]

Table S1: GLM results of seed final germination percentage (FGP) of the following factors: Seed conditions (stored or fresh), Temp (temperatures: 5, 10, 15, 20, 25, 25/10 and 30°C) and their interactions. Significance levels: *** $P < 0.001$, ** $P < 0.01$, * $0.01 \leq P \geq 0.05$, and ns: not significant ($P > 0.05$).

	**Residual deviance**	***F***	***P* (>F)**
Null	9.1977		
Seed conditions	235.58	7.053	< 0.001***
Temp	468.54	16.441	< 0.001***
Seed conditions × Temp	2.9124	-4.318	< 0.01**

Table S2: GLM results of viability of the following factors: Seed conditions (stored or fresh), Temp (temperatures: 5, 10, 15, 20, 25, 25/10 and 30°C) and their interactions. Significance levels: *** $P < 0.001$, ** $P < 0.01$, * $0.01 \leq P \geq 0.05$, and ns: not significant ($P > 0.05$).

	**Residual deviance**	***F***	***P* (>F)**
Null	3.7737		
Seed conditions	158.81	-0.277	0.783 ns
Temp	267.53	1.992	0.050*
Seed conditions × Temp	1.8584	-0.782	0.439 ns

Table S3: Mann-Whitney test results for leaf length and plant height means in both types of seed conditions (stored or fresh) by temperatures: 5, 10, 15, 20, 25, 25/10 and 30°C. Significance levels: *** $P < 0.001$, ** $P < 0.01$, * $0.01 \leq P \geq 0.05$, and ns: not significant ($P > 0.05$).

**Temperatures**	***P* (Leaf length)**	***P* (Plant height)**
5°C	0.013 *	0.017 *
10°C	0.033 *	0.050 *
15°C	0.215 ns	0.117 ns
20°C	0.010 *	0.011 *
25°C	0.003 **	0.001 ***
25/10°C	0.408 ns	0.535 ns
30°C	0.242 ns	0.145 ns

Table S4: Survival data of seedlings at the finish of the experiments (120 days). Seedlings were obtained from different seed conditions (stored or fresh) and different incubation temperatures (5, 10, 15, 20, 25, 25/10 and 30°C). *t*- test results for seedlings survival by seed conditions (stored or fresh). Data survival by seed category and temperatures were not analysed because there are not enough data. Significance levels: *** $\boldsymbol{P} < 0.001$, ** $\boldsymbol{P} < 0.01$, * $0.01 \leq \boldsymbol{P} \geq 0.05$, and ns: not significant ($\boldsymbol{P} > 0.05$).

**Temperature Conditions**	**% Stored Seed survival**	**% Fresh Seed survival**	***t*- test results**
5°C	87,50	62,50	-
10°C	70	100	-
15°C	100	100	-
20°C	100	90	-
25°C	100	70	-
25/10°C	75	87,50	-
30°C	60	80	-
**Mean**	**83,87**	**83,33**	$t = -0.0432$; $P = 0.966$ ns
